# Astrocyte-specific expression of interleukin 23 leads to an aggravated phenotype and enhanced inflammatory response with B cell accumulation in the EAE model

**DOI:** 10.1186/s12974-021-02140-z

**Published:** 2021-04-27

**Authors:** Louisa Nitsch, Simon Petzinna, Julian Zimmermann, Linda Schneider, Marius Krauthausen, Michael T. Heneka, Daniel R. Getts, Albert Becker, Marcus Müller

**Affiliations:** 1 Department of Neurology, University Clinic Bonn, Campus Venusberg 1, D-53127 Bonn, Germany; 2 Department of Surgery, University Clinic Bonn, Campus Venusberg 1, D-53127 Bonn, Germany; 3 Department of Neurodegenerative Disease and Geriatric Psychiatry, University Clinic Bonn, Campus Venusberg 1, D-53127 Bonn, Germany; 4 Department of Microbiology-Immunology and Interdepartmental Immunobiology Center, Northwestern University Feinberg School of Medicine, Chicago, USA; 5 Department of Neuropathology, University Clinic Bonn, Campus Venusberg 1, D-53127 Bonn, Germany; 6 School of Molecular Bioscience, University of Sydney, Sydney, Australia

**Keywords:** EAE, IL-23, Neuroinflammation, B cells, CNS

## Abstract

**Background:**

Interleukin 23 is a critical cytokine in the pathogenesis of multiple sclerosis. But the local impact of interleukin 23 on the course of neuroinflammation is still not well defined. To further characterize the effect of interleukin 23 on CNS inflammation, we recently described a transgenic mouse model with astrocyte-specific expression of interleukin 23 (GF-IL23 mice). The GF-IL23 mice spontaneously develop a progressive ataxic phenotype with cerebellar tissue destruction and inflammatory infiltrates with high amounts of B cells most prominent in the subarachnoid and perivascular space.

**Methods:**

To further elucidate the local impact of the CNS-specific interleukin 23 synthesis in autoimmune neuroinflammation, we induced a MOG35-55 experimental autoimmune encephalomyelitis (EAE) in GF-IL23 mice and WT mice and analyzed the mice by histology, flow cytometry, and transcriptome analysis.

**Results:**

We were able to demonstrate that local interleukin 23 production in the CNS leads to aggravation and chronification of the EAE course with a severe paraparesis and an ataxic phenotype. Moreover, enhanced multilocular neuroinflammation was present not only in the spinal cord, but also in the forebrain, brainstem, and predominantly in the cerebellum accompanied by persisting demyelination. Thereby, interleukin 23 creates a pronounced proinflammatory response with accumulation of leukocytes, in particular B cells, CD4+ cells, but also γδ T cells and activated microglia/macrophages. Furthermore, transcriptome analysis revealed an enhanced proinflammatory cytokine milieu with upregulation of lymphocyte activation markers, co-stimulatory markers, chemokines, and components of the complement system.

**Conclusion:**

Taken together, the GF-IL23 model allowed a further breakdown of the different mechanisms how IL-23 drives neuroinflammation in the EAE model and proved to be a useful tool to further dissect the impact of interleukin 23 on neuroinflammatory models.

**Supplementary Information:**

The online version contains supplementary material available at 10.1186/s12974-021-02140-z.

## Background

Multiple sclerosis (MS) is the most common autoimmune disease of the central nervous system (CNS) and the most common disabling neurological disease of the young adulthood. The key features of MS are infiltration of leukocytes, demyelinating plaques, and axonal damage [1]. Triggers and mechanisms leading to the development of the disease are not fully decoded, but it is widely accepted to be an autoimmune disorder with known genetic and environmental risk factors. Besides autoreactive T cells [2], B cells and plasma cells are essential components of MS lesions. B cell and plasma cell accumulation in perivascular lesions and in the subarachnoid space as well as leptomeningeal inflammation with ectopic lymphoid follicles were described in several studies [3–5]. The characteristic findings in the cerebrospinal fluid (CSF) of many MS patients are oligoclonal immunogobulin bands, which indicate intrathecal antibody synthesis. Thus, immunotherapies targeting B cells have been successful and approved in the treatment not only for relapsing-remitting MS patients but also for progressive MS patients [6].

Data of the pathogenesis and derived therapeutic approaches frequently arise from the experimental autoimmune encephalomyelitis (EAE) model as the animal model of MS. Although the EAE model was already discovered in the 1930s [7], it is still used to decipher the pathogenesis of autoimmune neuroinflammation until now. EAE can be induced with different kinds of myelin-derived antigens. One widely used is the myelin oligodendrocyte glycoprotein (MOG) 35-55 peptide. It is a CNS- specific myelin glycoprotein expressed on the outer surface of the myelin sheaths [8]. After immunization with the MOG35-55 peptide along with immune-activating adjuvants, the animals usually develop a monophasic course with an ascending paresis and subsequent regression of the symptoms over time.

With the help of the EAE model, it was possible to further decipher the role of cytokines in the development of MS. Cytokines are essential in the pathogenesis of various autoimmune disorders. Among the cytokines, a large number of studies have identified Interleukin 23 (IL-23) to play a critical role in various neuroinflammatory conditions, including MS [9–11]. It is part of the so-called Interleukin 12 (IL-12) cytokine family and consists of a unique p19 subunit and a common p40 subunit shared with IL-12 [12]. The IL-23 receptor complex consists of the IL-12Rβ1 component that binds to the p40 subunit and the specific IL-23 receptor (IL23R) subunit that binds to p19.

IL-23 is increased in serum and cerebrospinal fluid of MS patients [13, 14]. A significant increase of mRNA expression and protein production of both subunits of IL-23 was found in lesional compared to non-lesional tissues pointing to the possible local effect of IL-23 in MS [15]. In addition, in some MS patients, a single nucleotide polymorphism of the IL-23 subunit p40 increases the p40 expression and is associated with a younger onset of the disease and single nucleotid polymorphisms of the p19 subunit or the IL23R gene were associated with a higher risk of MS [9, 16]. Animal models emphasize the non-redundant role of IL-23 in the development of the EAE, as EAE cannot be induced in mice lacking IL-23 or the IL-23 receptor complex [17–19] and IL-23 produced by CNS-resident cells controls T cell encephalitogenicity during the EAE [17].

IL-23 is produced by classical antigen-presenting cells (APC) such as macrophages, dendritic cells, but can also be produced by astrocytes [12, 20, 21]. Activation of the IL-23 receptor complex leads to stimulation and activation of T helper 17 cells (Th17) lymphocytes. This subtype of CD4+ lymphocytes is crucial in chronic inflammation, autoimmune diseases and is detected in MS lesions [10].

Since naïve CD4+ cells lack the IL-23R, their differentiation into Th17 cells is initiated by the transformation of the growth factor β (TGFβ), IL-6 or IL-21, which consecutively stimulate the expression of the IL-23 receptor [22–24]. Upon IL-23 receptor signaling, Th17 cells produce various cytokines including their signature cytokines IL-17A, IL-17F, and others such as IL-21, IL-22, tumor necrosis factor a (TNFα), and colony stimulation factor 2 (GM-CSF).

To conclude, the critical role of IL-23 in neuroinflammation is striking. Nevertheless, the local impact of IL-23 in inflammatory CNS disorders such as MS remains elusive to some extent. To further characterize the impact of IL-23 on neuroinflammatory processes, we generated a transgenic mouse model with astrocyte-specific expression of IL-23 (GF-IL23). Our previous study showed that CNS-restricted IL-23-expression enables the spontaneous formation of infiltrates in the brain, especially in the cerebellum [24]. After several months, GF-IL23 mice develop an ataxic phenotype and cerebellar infiltrates with high amounts of lymphocytes particularly B cells. To further study the local influence of IL-23 in autoimmune CNS inflammation, we induced an EAE with MOG35-55 in the transgenic GF-IL23 mice. The aim of this project was to further clarify the effect of CNS-specific IL-23 expression on autoimmune neuroinflammation, especially concerning the impact of IL-23 on the disease course and the development of infiltrates, cell accumulation, and the proinflammatory cytokine milieu in EAE. We demonstrate that the local IL-23 production in the CNS leads to aggravation and chronification of the disease course with severe paraparesis and an ataxic phenotype. Local synthesis of IL-23 enhances gliosis and neuroinflammation not only in the spinal cord but also in the cerebellum, brainstem, and forebrain. The infiltrates mainly consist of lymphocytes with pronounced B cell accumulation. It leads to demyelination and induces a proinflammatory milieu with upregulation of cytokines and several complement factors. The data underline the importance of IL-23 in enhancing and maintaining autoimmune inflammation in the CNS.

## Methods

### Animals

The generation of transgenic mice with astrocyte-specific expression of particular genes was described in detail previously [24]. Genotyping of the transgenic offsprings was performed by polymerase chain reaction (PCR) of tail DNA with the primer previously published [24]. All mice were kept under standardized pathogen-free conditions at the animal facility of the University Hospital of Bonn, Germany. Animal experiments were approved by the Animal Care Commission of Nordrhein-Westfalen, Germany.

### Induction of EAE

Starting at the age of 6 months, GF-IL23 mice spontaneously develop an ataxia with cerebellar infiltrates [24], but neither clinical abnormalities nor histological changes occur before that age. To investigate the local effect of IL-23 in an EAE model, we immunized 2-month-old WT controls and transgenic animals at an age well before clinical symptoms and spontaneous neuroinflammation of the transgenic GF-IL23 mice occur. On day 0, mice were immunized subcutaneously into the rear flanks with a 1:1 emulsion of 100 μl MOG35–55 or bovine serum albumin (BSA) (3 mg/ml) in 100 μl complete Freund’s adjuvant (CFA) (Sigma-Aldrich, Munich, Germany) supplemented with 4 mg/ml Mycobacterium tuberculosis H37RA (Difco, Detroit, MI) as described before [25]. In addition, each mouse received an intraperitoneal injection of 500 ng pertussis toxin (Sigma-Aldrich, Munich, Germany) on days 0 and 2.

### Clinical assessment

Animals were weighed and examined for 60 days on a daily basis with two different scores: the typical EAE score [25] and the atypical score [26], reflecting an ascending paresis and an ataxic phenotype, respectively. The typical EAE score was assessed as follows: 0 no clinical symptoms, 1 limp tail, 2 hind limb weakness, 3 hind limb paralysis, 4 hind and forelimb paralysis, and 5 moribund. The atypical EAE score was assessed as follows: 0 no clinical symptoms, 1 hunched appearance, stiff tail, 2 staggered walking, scruffy coat, 3 head tilt, ataxia, obvious impaired balance/ambulation, body lean, 4 inability to maintain upright posture, severe axial rotation, severe body lean, and 5 moribund. Moribund mice with a score of 5 points were assigned to the score with the dominant clinical signs in the days before.

### Routine histology and immunohistochemistry

Mice were sacrificed by deep isoflurane anesthesia and transcardially perfused with phosphate-buffered saline (PBS). The brain was dissected and cut along the sagittal midline. After fixation with PBS-buffered 4% paraformaldehyde, half brains were embedded in paraffin or, for the cryosections, in Tissue Tek (Sakura Finetek, Staufen, Germany). The 10-μm paraffin-embedded sections were deparaffinized with xylene and rehydrated in graded ethanol series. Sections were stained with hematoxylin and eosin (HE) (Sigma-Aldrich, Munich, Germany) and Luxol fast blue (LFB) for routine histology and analysis of myelination, respectively. For immunohistochemistry, slides were incubated over night at 4°C with the primary antibodies (polyclonal rabbit anti-Laminin, Sigma-Aldrich, 1:50, polyclonal rabbit anti-GFAP 1:250) after incubation with Proteinase K (ThermoScientific, Waltham, USA; 1:1000) or incubated with biotin-conjugated tomato lectin (Axxora, 1:50) and washed with PBS. A corresponding biotinylated secondary antibody was used for 45min (Jackson ImmunoResearch, Newmarket, UK, 1:200) and horseradish peroxidase-coupled streptavidin for another 45 min (Vector Labs, Burlingame, USA; 1:200). For the immunoperoxidase, Nova Red (Vector Labs) as the substrate was used according to the manufacturer’s instruction. Sections were counterstained with hematoxylin (Sigma-Aldrich, Munich, Germany). For fluorescent immunohistochemistry, 10-μm cryosections were incubated with the primary antibodies (anti-CD3e BD Bioscience 1:500; anti-B220 BD Bioscience 1:200) over night at 4° C, washed with PBS, incubated with an A594 or A488 fluorescence-conjugated secondary antibody (Invitrogen, Darmstadt, Germany; 1:400) for 45 min, and counterstained with DAPI (Sigma-Aldrich, Munich, Germany).

A Nikon eclipse 800 bright-field and fluorescence microscope (Nikon, Duesseldorf, Germany) was used. Brightfield and monochrome fluorescent images were captured with a SPOT flex camera and SPOT advanced 4.5 software (Diagnostic instruments, Sterling, USA). Myelinated and demyelinated areas of the spinal cord were measured on cross-sections with ImageJ image analysis software to determine the proportion of the demyelinated spinal cord area.

### Flow cytometry

Isolation of microglia and infiltrating leukocytes from the cerebellum was described in detail before [27]. Mice were sacrificed by deep isoflurane anesthesia and transcardially perfused with 4°C cold 1x PBS for removal of intravascular leukocytes until the fluid was clear. The dissected cerebellum and myelon were homogenized in Hank’s Balanced Salt Solution (HBSS, Gibco, Eggenstein, Germany) by a tissue homogenizer (glass potter, Braun, Melsungen, Germany) before passing through a 70-μm cell strainer (BD biosciences, Heidelberg, Germany). After centrifugation, the pellets of the homogenates were resuspended in 75% isotonic 4°C Percoll solution (GE-Healthcare, Uppsala, Sweden) and overlayed with 25% and 0% isotonic Percoll solution. The Percoll density gradient was centrifuged for 25min, 800g at 4°C. From the 25%/75% Percoll solution interface microglia, leukocytes and astrocytes were removed. After washing with 1xPBS, cells were blocked with CD16/CD32 antibody (Fc block; eBioscience, Frankfurt/Main, Germany) and incubated with fluorochrome-conjugated antibodies (eBioscience; BD Bioscience, Heidelberg, Germany) for the detection of CD3e, CD4, CD8a, CD11b, CD19, CD45, NK1.1, Ly6G, and γδTCR. Acquisition was performed with a BD FACSCanto II flow cytometer (BD Biosciences, Heidelberg, Germany). Data were analyzed using the flow cytometry software FlowJo (TreeStar, San Carlos, CA).

### Transcriptome analysis

mRNA of cerebellum homogenates (WT/transgenic mice) was isolated using Trizol reagent (Invitrogen) according to the manufacturer’s instructions. Library generation for 3′-mRNA sequencing was conducted according to manufacturer’s guidelines (Quant Seq 3′ mRNA-Seq Library Prep Kit FWD for Illumina; Lexogen). Briefly, 100-ng total RNA was reversely transcripted by oligo-dT-priming (first strand) and random priming (second strand). After a magnetic bead-based purification step, the libraries were amplified using 15 polymerase chain reaction cycles. Libraries were sequenced on a HiSeq 2500 v4 with a read length of 1×50bp. On average, 10 million raw reads were generated per sample. After trimming of Illumina Universal Adapter with cutadapt, reads have been aligned to the mouse genome (STAR). FeatureCounts was used to assign reads to genes based on the definitions of ENSEMBL release GRCm38.94. Inclusion criteria for counting were as follows: uniquely mapped, matching strand, and overlap with only one gene, i.e., non-ambiguous assignment to a single gene. Statistical analysis was performed in R environment (version 3.5.2) with the Bioconductor R-package DESeq2 with *p*<0.05 considered to be significant. Only genes with a total sum of at least 2 counts across all samples were considered for the analysis. Data visualization, such as heatmaps, was generated upon variance stabilizing transformation transferred data using Complexheatmaps. To generate functional categories of upregulated mRNA levels, targets from the data set that met the log2 fold change >1 cutoff and an *p* value < 0.05 were uploaded to the Database for Annotation, Visualization and Integrated Discovery (DAVID). Relevant categories of cellular functions and pathways were depicted [28, 29]. The number of targets from the data set mapping to the category is displayed. A modified Fisher’s exact test was used to calculate a *p* value determining the probability that the association to the category is by chance alone with *p* <0.05 considered to be significant. Only significant categories were displayed.

### q-PCR

mRNA was isolated using Trizol reagent (Invitrogen, Darmstadt, Germany). For transcription into cDNA, 1-μg total mRNA was used using SuperScript™ III Reverse Transcriptase (Invitrogen). Real-time quantitative PCR assays were performed using Taqman reagents for the following targets TNFα, IL-1b, IL-17a, IFNγ, CXCR3, and CD68 (Applied Biosystens, Darmstadt, Germany) for transgenic and WT mice (*n*=5). Targets were analyzed in duplicates. Mean mRNA level was normalized to the mRNA level of GAPDH as the internal control.

### Statistical analysis

Data of the clinical assessment were analyzed where appropriate by a Mann–Whitney *U* test or a logrank test with a statistical significance defined as *p < 0.05, **p < 0.01, and ***p < 0.001. The statistical analysis of the flow cytometry and qPCR data were was performed using a two-way ANOVA followed by an appropriate post hoc test with *p < 0.05, **p < 0.01, and ***p < 0.001. All statistical analyses were performed using GraphPad Prism 5.0 (GraphPad Software).

## Results

### CNS-restricted expression of IL-23 leads to a severe and chronic course of the EAE

To further investigate how the CNS-restricted expression of IL-23 influence the clinical course of the EAE-model as an animal model of MS, GF-IL23 mice and WT were immunized with MOG35-55 at an age of 2 months.

**Fig. 1:**
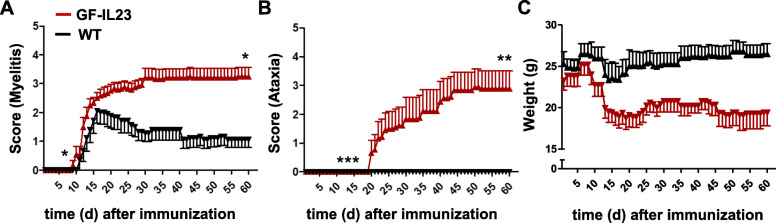
Exacerbation of EAE in GF-IL23 mice. **a**, **b** GF-IL23 mice develop a more severe EAE with an ascending paralysis and in addition an ataxic phenotype compared to WT mice. The characteristic findings are also summarized in Table 1. **c** Consistent with the clinical phenotype the GF-IL23 mice show a considerable weight loss compared to the WT mice. The data represent 9-12 GF-IL23 mice and 9 WT mice

The WT mice developed a typical EAE course with a progressive paraparesis with a disease onset at day 15.5 (± 7.5) and the maximum score at day 23.5 (± 14.0) (Tbl.1, Fig. 1a). They exhibited a peak/maximum score of 2.5 (± 0.4) points measured on the common EAE scale for the ascending paresis with incomplete recovery of the deficits until day 60 with an average score of 1.1 (± 0.3).

**Table 1:** Clinical features of the EAE in GF-IL23 and WT mice

	**Myelitis**		**Ataxia**	
	GF-IL23	WT	GF-IL23	WT
**Incidence**	12/12	8/9	7/9	0/9
**Disease (days) onset**	11.5 ± 1.5*	15.5 ± 7.5	36.0 ± 14.5***	-
**Time (days) to maximum**	21.5 ± 6.0	23.5 ± 14.0	44.5 ± 10.0***	-
**Maximum score**	3.3 ± 1.0	2.5 ± 0.4	2.9 ± 1.8**	-
**Outcome (d60)**	3.3 ± 1.0***	1.1 ± 0.3	2.9 ± 1.7**	-

*p < 0.05, **p < 0.01, ***p < 0.001

However, GF-IL23 mice showed an earlier onset of the progressive paraparesis (day 11.5 ± 1.5) compared to WT mice, but also developed a more severe, albeit not significant, course with an average maximum score of 3.3 (± 1.0) points. In contrast to the WT controls, the transgenic mice did not recover from the deficits, but rather exhibited a chronic disease with an outcome score of still 3.3 (± 1.0) points.

Beside the progressive paraparesis, GF-IL23 mice developed a progressive ataxic phenotype, which prompted us to use an additional score for ataxia to monitor these mice more accurately. The ataxia started at an average at day 36.0 (± 14.5) after immunization with a maximum score of 2.9 (± 1.8) on day 44.5 (± 10.0) without a relevant recovery until day 60 (Tbl. 1, Fig. 1b). In comparison, none of the WT controls developed an ataxia. Correlating with the more severe clinical manifestation, GF-IL23 mice showed a pronounced weight loss compared to WT mice (Fig. 1c). In contrast, WT and transgenic controls immunized with only BSA showed neither clinical deficits (data not shown) nor histological signs of infiltration in cerebellum, myelon, or upregulation of proinflammatory marker (Fig. S1).

To summarize, CNS-specific expression of IL-23 led to a chronic progressive EAE with a severe form of the ascending paresis and, in contrast to WT controls, a severe ataxia without a regression of the deficits.

### Enhanced spinal cord infiltration in GF-IL23 mice and persisting demyelination

For the following experiments, 5 different time points, days 0, 15, 22, 33, and 60, were depicted based on the clinical course of the EAE. Thereby, day 15 represents the analysis at the beginning of the disease, day 22 the experiments shortly after the maximum score of the ascending paresis and before onset of the ataxia, day 33 the period around the onset of the ataxia, and day 60 the results in the chronic stage and at the end of the observation period.

**Fig. 2:**
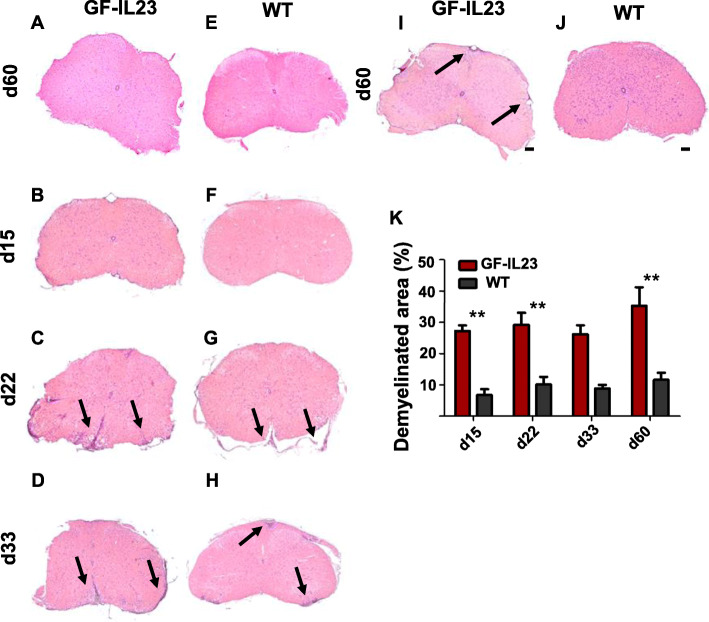
Increased myelon infiltration in the cerebellum of EAE-induced GF-IL23 mice. HE-staining of the myelon (transverse sections) at different time points (d0, d15, d22, d33, d60). GF-IL23 naïve mice show no infiltration (**a** WT control E). **b**–**d** I GF-IL23 mice show enhanced infiltration over the time, especially at d22, d33 compared to WT mice (**f**, **g**, **h**, **j**). Arrows point to infiltrates. Scale bar: 100 μm. The images are representative of those from at least different 8 transgenic/WT mice d0, d15, d22, d33, and 8 transgenic, 6 WT mice d60. **k** Demyelinated area relative to the white matter area was quantified in cross-sections of the spinal cord from GF-IL23 and WT mice (*n* = 4–5 for each group) with *p < 0.05 and **p< 0.01 and showed a significant enhanced demyelination in GF-IL23 mice

The myelon of the EAE transgenic animals displayed pronounced infiltrates in transverse sections around the period of the maximum score on day 22, but also up to day 33 compared to WT mice (Fig. 2a–h). In the chronic stage, at day 60, the EAE GF-IL23 mice show only few infiltrates, but still pronounced demyelination compared to WT mice (Fig. 2i–k).

### Immunization of GF-IL23 mice with MOG35-55 peptide results in prominent infiltration of the cerebellum, brain stem, and forebrain

**Fig. 3:**
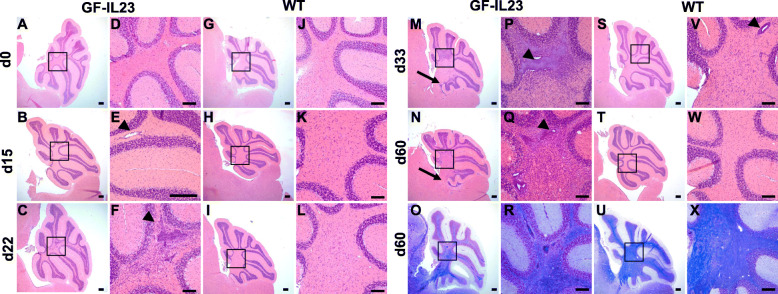
Subarachnoidal and intraparenchymal infiltration in cerebellum of EAE-induced GF-IL23 mice. HE- and LFB-staining of the cerebellum at different time points (d0, d15, d22, d33, d60) after induction of the EAE. Naïve GF-IL23 mice show no infiltration (**a** higher magnification (**d**), WT controlls (**g**, **j**)). GF-IL23 mice (**b**, **c**, **m**, **n**, **o** higher magnification (**e**, **f**, **p**, **q**, **r**)) show increasing subarachnoidal (arrow) and intraparenchymal (arrow head) infiltrates over the time and persistent demyelination on day 60 whereas WT mice (**h**, **i**, **s**, **t** higher magnification (**k**, **l**, **v**, **w**)) show only little and few infiltrates without persisting demyelination on day 60. Scale bar: 100 μm. The images are representative of those from at least 8 different transgenic/WT mice d0, d15, d22, d33, and 8 transgenic, 6 WT mice d60

**Fig. 4:**
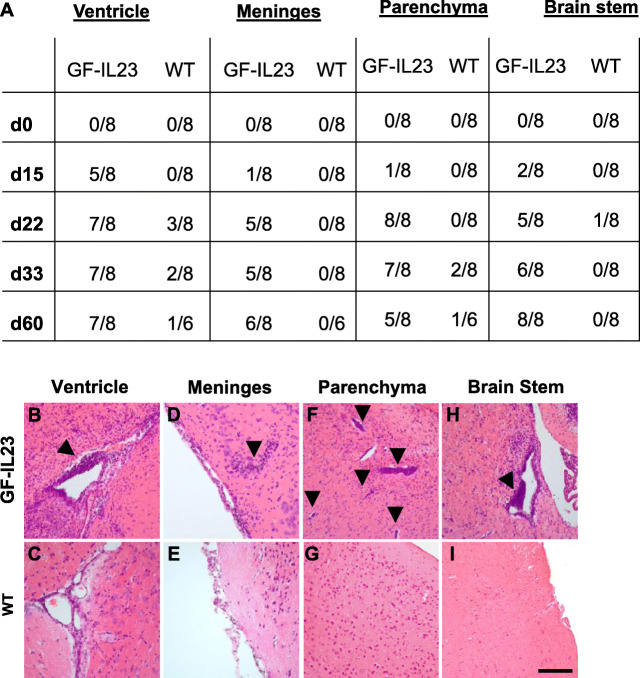
Infiltration of the ventricle, meninges, and parenchyma of the forebrain and brain stem in GF-IL23 mice. **a** Fraction of GF-IL23 and WT EAE mice with cellular infiltrates in ventricles, meninges, parenchyma, or brain stem. Naïve mice did not show these infiltrates. Transgenic mice show infiltrates from day 15 in these locations, whereas only sporadic infiltrates were found in WT mice. **b**–**i** Exemplary histology findings at d33. **b**, **d**, **f**, **h** Beside the cerebellum, infiltrates (arrow heads) in other locations such as ventricle, meninges, and parenchyma of the forebrain and brain stem were found in GF-IL23 mice. WT mice (**c**, **e**, **g**, **i**) display no infiltrates in these locations. Scale bar: 100 μm. The images are representative of those from at least 8 different transgenic/WT mice d33

**Fig. 5:**
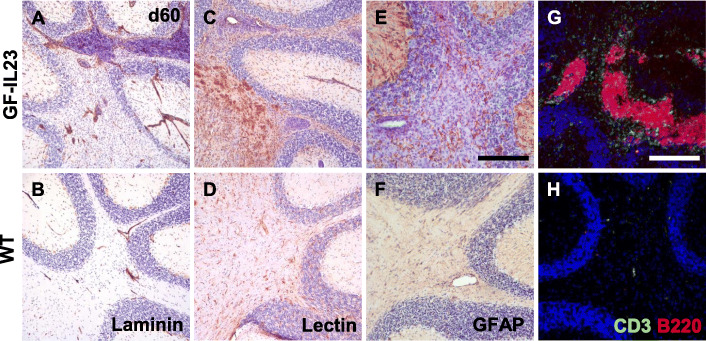
Hypervascularization, microglia, astrocyte activation, and B cell accumulation in GF-IL23 cerebellum. Histology findings at d60. **a**–**f** GF-IL23 mice display hypervascularization (**a**), microglia, and astrocyte activation (**c**, **e**) compared to WT mice (**b**, **d**, **f**). **g**, **h** Fluorescent immunohistochemistry of GF-IL23 cerebellum reveals that the infiltrates consist of large amounts of B cells. Scale bar: 100 μm. The images are representative of those from at least 8 different transgenic/6 WT mice

Congruent with the ataxic phenotype, the cerebellum of EAE GF-IL23 mice displayed pronounced infiltrates in the histology. Naïve mice show no inflammatory infiltrates (Fig. 3a, d, g, j). On day 15, even before onset of the ataxia, smaller infiltrates became manifest, especially intraparenchymal and perivascular in transgenic mice (Fig. 3b, e). On days 22, 33, 60 along with the clinical manifestation of the cerebellar ataxia, there is a stronger infiltration including also subarachnoidal infiltrates at this stage (Fig. 3c, f, m, n, p, q). WT showed only mild and sporadic infiltrates (Fig. 3h, I, k, l, s, t, v w). Accompanying the infiltrates, the GF-IL23 mice showed pronounced demyelination in the tissue surrounding the infiltrates (Fig. 3 o, r, u, x). Beside infiltration of the myelon and cerebellum, GF-IL23 mice developed a widespread CNS infiltration with ventricular and meningeal involvement, smaller intraparenchymal infiltrates in the forebrain and in the brain stem (Fig. 4a, b, d, f, h). WT mice, on the other hand, showed no or only few small infiltrates in these regions (Fig. 4a, c, e, g, i). Immunohistochemistry of the cerebellum revealed microglia and astrocyte activation in the transgenic immunized animals compared to WT mice (Fig. 5c–f for d60, Fig. S2 for d0, d15, d22, d33). In addition, hypervascularization was detected on day 60 (Fig. 5a, b).

### Infiltrates consist mainly of lymphocytes with a high proportion of B cells

The infiltrates were further characterized by immunohistochemistry and flow cytometry analysis. Immunofluorescence histology of the cerebellum illustrated that the infiltrates of the transgenic mice predominantly consist of lymphocytes with a high proportion of B cells (Fig. 5g, h for d60, Fig. S2 for d0, d15, d22, d33), which was confirmed by flow cytometry data as well.

**Fig. 6:**
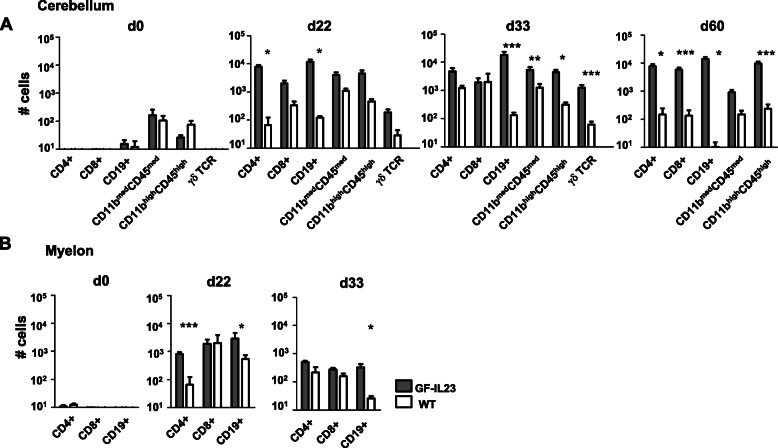
Infiltrates consist of large amounts of B cells. **a** Flow cytometry data of the cerebellum (absolute numbers) on days 0, 22, 33, and 60 show consistent findings with predominant CD19+ cells in the cerebellum of MOG-immunized GF-IL23 mice. In addition, CD4+ cells (d22, d60), CD8+ cells (d60), CD11b^high^CD45^high^ (d33, d60), and also γδ TCR+ cells (d33) were elevated compared to WT mice. **b** Flow cytometry data of the myelon on day 22 and day 33 show elevation of CD4+ and CD19+ cells on d22 and CD19+ cells compared to WT mice. The mean value, standard deviation are given with *p < 0.05, **p< 0.01, ***p< 0.001 with *n*=5 transgenic/WT mice d0, *n*= 8–12 transgenic/WT mice d22, d33, and 6 transgenic/4 WT mice d60

In the cerebellum of immunized GF-IL23 mice, there was an increase of CD4+ cells on day 22 and d60 and of CD8+ cells on day 60 (Fig. 6a). Beside the T cell infiltration, vast amounts of CD19+ B cells were detectable in the cerebellum of GF-IL23 mice at day 22, d33, d60. Non-immunized transgenic mice did not show lymphocyte infiltration. Regarding other immune cell populations, an increase of CD11b^high^ CD45^high^ cells could be detected on d33 and d60. γδ T cells were elevated on day 33. NK 1.1+ and Ly6G+ cells were not elevated (data not shown).

In the myelon, CD19+ B cells were elevated on day 22 and day 33 and CD4+ cells on day 22 compared to WT mice (Fig. 6b). Other cell populations, such as NK 1.1 and γδ T cells were not elevated (data not shown).

Taken together, the flow cytometry data demonstrated a pronounced infiltration of lymphocytes, particular B cells next to CD4+, CD8+ cells in the cerebellum and CD4+ cells in the myelon accompanied by γδ T cells on day 33 and CD11b^high^ CD45^high^ cells on day 33 and 60 in the cerebellum of the transgenic mice.

### IL-23 induces cell activation, a proinflammatory cytokine profile, and upregulation of complement components

**Fig. 7:**
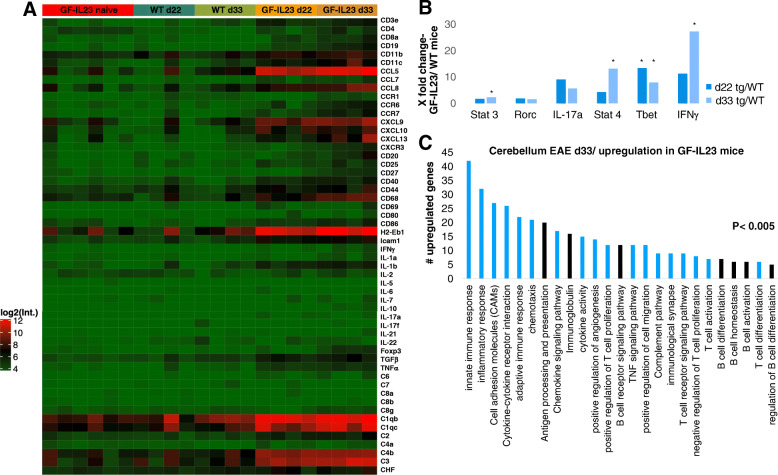
mRNA-profile of the cerebellum. **a** The transcriptome analysis illustrates the mRNA levels of several surface cell markers, pro-/antiinflammatory markers, and complement components of the cerebellum of GF-IL23 and WT mice at d22 and d33 after induction of the EAE. **b** The mRNA levels of targets involved in Th17 and Th1 cell signaling are listed from MOG-immunized mice at d22 and d33 in relation to WT immunized with *p < 0.05. **c** To generate functional categories of upregulated mRNA levels, targets from the data set that met the log2 fold change >1 cutoff and an *p* value < 0.05 were uploaded to the Database for Annotation, Visualization and Integrated Discovery (DAVID) and relevant categories of cellular functions and pathways were depicted. The number of targets from the data set that map to the category is displayed. Only significant categories with *p* <0.05 were displayed. In black, the signaling pathways associated with B cells are depicted. Data were generated from naïve GF-IL23, *n*=6, MOG-immunized GF-IL23/WT mice, *n*=4. CD cluster of differentiation, CCL chemokine (C–C motif) ligand, CCR C–C chemokine receptor type, CXCL chemokine (C–X–C motif) ligand, H2-Eb1 histocompatibility 2, class II antigen E beta, ICAM-1 intercellular adhesion molecule 1, IFNγ interferon gamma, IL interleukin, Foxp3 forkhead box P3, C1-8 complement component 1-8, CFH complement component factor H, Stat signal transducer and activator of transcription, Rorc RAR-related orphan receptor C, T bet T box expressed in T cells

For the analysis of surface cell markers, activation markers, inflammation mediators, and complement factors, a transcriptome analysis of the cerebellum of the transgenic and WT animals was performed (Fig. 7a, for the statistical parameters Fig. S3). The findings with elevated mRNA levels of the surface cell marker CD19 at day 22 and day 33 were in line with the flow cytometry results. Furthermore, the expression of CD4 on d22 and d33, CD8 on day 33, and CD11b on day 33 was also significantly increased in the transgenic mice as was the expression of CD11c.

The expression of other surface cell markers, among them markers for T and B cell activation and co-stimulatory molecules, such as CD20, CD25, CD40, CD27, CD44, CD68, CD69, CD86, ICAM-1, and H2-Eb1, was increased.

In addition, the mRNA levels of various proinflammatory cytokines and cytokine receptors were elevated in the transgenic cerebellum such as CCL5, CCL7, CCL8, CCR6, CCR7, CXCL9, CXCL10, CXCL13, CXCR3, IFNγ, IL-1a, IL-1b, TNFα, and TGFβ, but not significantly the Th17 cell signature cytokines IL-17a, IL-17f, IL-21, and IL-22. Foxp3, playing an important role in regulatory T cell function, was also elevated.

Furthermore, analysis of the complement system indicates increased expression of C1qb, C1qc, C2, C3, and C4b on day 22 and 33 as well as C4a on day 33 and complement component factor H (CFH) on day 22.

Figure 7b lists the expression of the typical downstream targets involved in Th17 and Th1 cell signaling in GF-IL23 compared to WT mice. Regarding Th17 cell signaling, signal transducer and activator of transcription (Stat) 3 was elevated in d33 transgenic mice, but not IL-17a or RAR-related orphan receptor C (Rorc), which encodes RAR-related orphan receptor gamma (Rorγ). In d22 and d33 mice, T box expressed in T cells (T bet), in d33 mice Stat 4 and IFNγ, all involved in Th1 cell signaling, was upregulated in GF-IL23 mice.

Furthermore, we generated functional categories of upregulated mRNA targets in immunized GF-IL23 mice compared to WT immunized mice and relevant categories of cellular functions and pathways were depicted and illustrated in Fig. 7c. We found categories of the innate and adaptive immune response are important in our model. But also upregulation of cell adhesion molecules, targets involved in chemotaxis, antigen processing and presentation, and B and T cell activation/proliferation/differentiation are relevant in the EAE GF-IL-23 model.

In summary, the transcriptome analysis also illustrates a B and T cell accumulation in the cerebellum. But it also points toward T and B cell activation, upregulation of co-stimulatory molecules, proinflammatory cytokines, and components of the complement system.

## Discussion

MS is an autoimmune disorder resulting in focal neuroinflammatory lesions in the gray and white matter. Besides neuroinflammation, demyelination, and axonal damage throughout the brain are the hallmarks of MS. IL-23 has been established as one of the crucial cytokines in autoimmune disorders and especially in the pathogenesis of MS and corresponding animal models [9, 13, 14, 17].

Our previous study has illustrated that CNS-restricted IL-23-expression induces the spontaneous formation of infiltrates in the brain, especially in the cerebellum [24]. These GF-IL23 mice spontaneously develop a progressive ataxic phenotype, which corresponds to cerebellar tissue destruction and inflammatory infiltrates most prominent in the subarachnoid and perivascular space. The CNS-cytokine milieu was characterized by elevation of numerous inflammatory mediators such as IL-17a and IFNγ. However, the leukocytic infiltrates were surprisingly predominated by B cells. In the current study, GF-IL23 mice and WT controls were immunized with MOG 35-55 to evaluate the impact of locally synthesized IL-23 on the course of EAE as the animal model of MS.

After immunization with the peptide MOG 35-55, the transgenic mice showed a slightly earlier onset of the progressive paraparesis compared to WT mice. Furthermore, the GF-IL23 mice developed an ataxia and most essentially no remission of both paraparesis and ataxia. This indicates that CNS-specific synthesis of IL-23 is not only crucial for the development of the paraparesis and ataxia, but also leads to a rather chronic course of the EAE.

In addition, the GF-IL23 mice developed a pronounced stronger infiltration in the spinal cord with marked reduction of infiltration at the end of the observation period. In contrast, severe clinical deficits persisted in the presence of IL-23. The ongoing para- or tetraparesis appeared to be related to the stronger demyelination on day 60, which was more pronounced in GF-IL23 mice. The clinical and histological findings of the spinal cord point toward a direct or indirect role of IL-23 in demyelination and interfering with remyelination after EAE induction. Demyelinated lesions are one of the hallmarks of MS and are present in the white as well as in the gray matter [30]. Remyelination is a crucial step during the remission phase of an MS relapse as it preserves the axon from dissection and degeneration. In chronic MS, incomplete spinal cord remyelination correlates with higher disease-related disability [31].

Moreover, besides the clinical and histological signs of spinal cord affection, the cerebellum of the GF-IL23 mice showed a chronic EAE course with manifest and here persisting infiltration and again pronounced demyelination. In addition, infiltrates were detected at various other CNS locations such as ventricles, meninges, in the forebrain, and brain stem.

The inflammatory response of GF-IL23 mice during EAE is a specific response to the immunization with MOG-peptide, because BSA-immunized GF-IL23 mice did not show any of the described pathological features. In our previous study, although we saw infiltrates in the long-term LPS experiment in GF-IL23, these animals did not show an ataxia, the extent of infiltrates that we see in EAE or these multifocal infiltrations [24].

There is evidence that IL-23 have a greater impact on the onset of autoimmune CNS inflammation than on the maintenance of the inflammatory response. A prophylactic effect of anti-p40 antibody treatment has been shown in a myelin-induced EAE model in marmosets [32]. The treatment during the ongoing disease with anti-IL-12p40 antibodies delays the clinical manifestation of existing lesions. However, once neurological deficit has become manifest, progression to serious deficits seems less influenced by anti-IL-12p40 treatment [33]. One possible explanation for the failure of the anti-p40 antibody ustekinumab phase II clinical trial in relapsing–remitting MS is that the possibility for a beneficial treatment might have already passed once the patients were enrolled [34]. Thakker et al. showed that encephalitogenic T cells from MOG-immunized WT mice caused indistinguishable disease when adoptively transferred to WT or IL-23-deficient recipient mice, demonstrating that once encephalitogenic cells have been generated, EAE can develop independent from IL-23 [35]. In contrast to these findings, the course of EAE in GF-IL23 mice observed in our study argues for a role of local IL23 beyond the disease onset. In the GF-IL23 mice, the EAE is chronic and not monophasic as in the WT control, so astrocyte-specific expression of IL-23 appears to play a role in the maintenance of neuroinflammation. The progressive infiltration suggests that IL-23 not only triggers the inflammation, but in addition also contributes to the further course in our model.

In comparison to other mouse models with astrocyte-specific expression of certain cytokines, the disease course as well as the histochemistry data with the B cell infiltration of the GF-IL23 mice in the EAE model is quite remarkable and emphasizes the findings in our study are IL-23-specific. After immunization with MOG35-55, GF-IL12 mice with astrocyte-specific expression of IL-12 had an earlier onset and higher incidence but not the chronic EAE course and ataxia, nor the multilocular inflammation or pronounced tissue destruction seen in GF-IL23 mice [36]. By contrast, MOG35-55-immunized GF-IL6 mice developed a severe ataxia, but no signs of spinal cord involvement [25]. Infiltration and demyelination were nearly absent from the spinal cord, but significantly increased in the cerebellum of EAE GF-IL6 mice with accumulation of neutrophils and B cells accompanied by tissue damage. Thus, site-specific production of IL-6 in the cerebellum redirects the leukocytes away from the normally preferred antigenic site of the spinal cord with markedly enhanced inflammatory cell accumulation in the cerebellum [25]. This seems to be different to the GF-IL23 model. Here, the CNS-specific IL-23 expression leads to a strong and chronic neuroinflammation in the EAE model with high amounts of B cells and to a multilocular inflammation, thus particularly pronounced in the spinal cord and cerebellum.

IL-23 is produced by APC such as macrophages or dendritic cells [12, 20], but has also been detected in astrocytes [21]. Studies demonstrate that local production of IL-23 in the CNS [17, 18] controls T cell encephalitogenicity in EAE. However, regarding the GF-IL23 model, some points need to be considered. In the GF-IL23 modell, IL-23 overexpression occurs only in astrocytes. Furthermore, IL-23 secretion is at the beginning of the neuroinflammatory process during EAE in the GF-IL23 model and is not secondary to immunization with MOG as in WT EAE mice. In the GF-IL23 model, astrocyte-specific secretion of IL-23 persists, independent of the immunization with MOG. Therefore, the data on EAE in GF-IL23 cannot reflect all aspects of IL-23 in autoimmune neuroinflammation, but may be able to further study some aspects such as the B cell role in the IL-23 mediated neuroinflammation.

Immunohistochemistry of the cerebellum, flow cytometry data of the myelon and cerebellum, and data from the transcriptome analysis of the cerebellum showed that the infiltrates of the GF-IL23 mice predominantly consisted of lymphocytes. In the spinal cord, an increase of CD19+ cells and CD4+ cells were detected. In the cerebellum, the flow cytometry results with elevated T cells, especially CD4+ cells, and CD19+ cells were even more pronounced. The transcriptome analysis revealed elevated levels of Foxp3 during the course of the EAE in the cerebellum pointing to also regulatory immune mechanisms during the disease course.

The dominant B cell accumulation during EAE in mice with a C57BL/6 genetic background is a very uncommon finding, as this EAE model is usually a mainly T cell-driven model of CNS inflammation [37]. But compared to our previous findings describing the spontaneous course of the GF-IL23 mice, the B cell accumulation during EAE in GF-IL23 mice is less surprising and underlines a role of IL-23 in attracting and accumulating B cells into the subarachnoidal space and into the CNS parenchyma during neuroinflammation [24]. In the EAE model, the GF-IL23 mice developed these infiltrates accelerated and much earlier after the immunization with MOG 35-55 peptide.

In healthy humans, only very few B cells are present in the CNS parenchyma and CSF, where they represent less than 1% of leukocytes [38, 39]. Though, in patients with CNS inflammation, B cell numbers can rise and accumulation of B cells is one of the characteristic findings in MS. Histopathological studies of the brain tissue from progressive MS patients revealed leptomeningeal inflammation with ectopic lymphoid follicles, B cells and plasma cells. In relapsing-remitting MS, B cell accumulation in the CNS was also demonstrated [5, 40]. B cell rich meningeal inflammation was associated with increased spinal cord pathology in secondary progressive MS patients [41]. In general, B cells are enriched in perivascular lesions and in the subarachnoid space in these patients, but are also sparsely present in the CNS parenchyma [3–5]. The central role of B cells in the pathogenesis of MS was further substantiated by the beneficial and approved treatment of relapsing-remitting and active primary progressive MS patients with B cell depleting therapies [6, 42, 43].

The B cell infiltration upon CNS-specific IL-23 expression in our model is impressive. The mechanisms by which these B cells contribute to the phenotype can be various. B cells modulate CNS inflammation by humoral and cellular mechanisms like antibody secretion, cytokine release, as antigen-presenting cells by interaction with and co-stimulation of T cells [44–46]. The transcriptome analysis showed evidence for T and B cell activation, proliferation and differentiation and the role of co-stimulatory surface antigens in the GF-IL23 model during EAE. Surface cell markers for T cell activation such as CD25, CD44, and CD69 and markers for B cell activation such as CD86 were clearly elevated. Other co-stimulatory molecules such as CD27 as a regulator of B cell activation and immunoglobulin synthesis and CD40 were also elevated. CD40 is expressed on B cells and upon binding to the CD40L, expressed on activated CD4+ cells, leads to B cell proliferation, memory B cell generation, and antibody class switching [47]. Binding of T cells to CD40 and CD86 on the other hand leads to activation and differentiation of encephalitogenic T cells. Antigen processing and presentation plays also a role in the transgenic EAE mice. The high level of histocompatibility 2, class II antigen E beta (H2-Eb1) chain, which encodes the Major histocompatibility complex class II (MHC II) protein complex, is striking in our model. MHC II is generally expressed on APC and presents antigens to CD4+ cells. Among the APCs, B cells are highly efficient in presenting antigens even in low antigen concentrations as they can recognize CNS antigens by their specific receptor [48, 49]. The MHC class II-restricted antigen presentation by B cells is essential for the induction of EAE that require both B cells and T cells. It allows B cells to present myelin and other CNS antigens to encephalitogenic T cells [50]. An EAE cannot be induced in mice with selective MHCII-knockout in B cells, and these mice show a diminished Th1 and Th17 cell response [51, 52]. Furthermore, the upregulation of ICAM-1 in our study is quite interesting since ICAM-1 induced in response to TNFα, IL-1β, or IFNγ mediates T cell migration across the blood-cerebrospinal fluid barrier and blood brain barrier and is expressed on antigen-presenting cells [53].

Various proinflammatory cytokines and cytokine receptors including IL-1a, IL-1b, the T helper 1 (Th1) signature cytokine IFNγ, and TGFβ, TNFα were elevated indicating a pronounced proinflammatory milieu in the cerebellum of the transgenic mice due to the local IL-23 synthesis. Surprisingly, Th17 signature cytokines IL-17a, IL-17f, IL-21, and IL-22 were not significantly elevated when compared to WT mice. Consistent with this, the Th17 cell transcription factor Rorc is also not upregulated, whereas Stat3 is upregulated in d33 transgenic mice. This indicates that the IL-23 mediated mechanism of immune cell accumulation and aggravation of the local proinflammatory cytokine milieu may be partly independent of the Th17 cell axis. In our study, we found elevation of the Th1 cell-associated Stat4, Tbet besides IFNγ in GF-IL23 mice during EAE. This is in line with our previous study of the spontaneous course of the GF-IL23 mice. There we found increased levels of IFNγ and also Th1 cells in the inflammatory cerebellum, but also IL-17a and IFNγ co-expressing CD4+ cells in older mice [24]. In MS patients, the levels of IFNγ are associated with the disease activity [54] and the myelin-reactive T cell repertoire produces IFNγ in response to antigens [55]. By increasing the expression of MHC molecules, IFNγ as well enhances the antigen-presentation of myeloid cells, which in turn results in activation of myelin-antigen-specific CD4+ or CD8+ cells [54]. We could speculate that upregulation of B/T cell co-stimulatory molecules in the GF-IL23 EAE model mentioned above makes T cells more active and aggressive, leading to the increased expression of IFNγ.

In addition, the findings of the transcriptome analysis in our model point to a role of IL-23 in enhancing the EAE driven chemokine response. CCL5, CCL7, CCL8, CXCL9, and CXCL10 secretion is IFNγ, TNFα, and IL-1 dependent [56, 57]. It should be noted that all of these cytokines, IFNγ, TNFα, and IL-1 are pronounced elevated in the GF-IL23 mice and are therefore likely responsible for the upregulation of these chemokines. CCL5 is secreted by various cell types and can attract T cells, monocytes, basophils, eosinophils, NK cells, dendritic cells, and mast cells [58]. In the CSF of MS patients, higher levels of CCL5 and CXCL10 were detected compared to healthy controls [59]. It is postulated that T cells, able to transmigrate into the CSF, express CXCR3 mostly independently of a possible inflammatory CNS condition, but only in the presence of the CXCR3 ligand CXCL10 in the CSF they remain in the CSF and do not recirculate into the periphery [60]. Another chemokine, CXCL13, which is intimately involved in B cell trafficking [61], was highly elevated during EAE of the GF-IL23 mice. CXCL13 is increased in the meninges and CSF of postmortem MS cases with high levels of meningeal inflammation and demyelination. Furthermore, elevated CSF levels correlate with B cell count in the CSF, markers of immune activation, disease activity, and gray matter damage [62, 63]. The role of CXCL13 was further substantiated since the level of CXCL13 in the CSF correlates with clinical response to B cell-depleting therapies in MS patients [64].

Furthermore, the findings concerning the complement system with increased expression of several components such as C1qb, C1qc, C2, C3, C4a, C4b, and CFH in the EAE of GF-IL23 mice are intriguing. In some MS patients, autoantibodies contribute to tissue destruction by binding to the surface of brain cells and by attacking them dependent on complement factors [65]. The importance of the complement system in the EAE has been demonstrated in several studies [66–68]. Hundgeburth et al. described the contribution of the complement system to the demyelination and modification of the T and B cell response in the EAE model. Mead et al. also proved the impact of the complement system on demyelination in the EAE model. In addition, there is one study, which directly links IL-23 to the complement system [69]. In this study, they analyzed an EAE model in the absence of the cell surface C3/C5 convertase inhibitor decay-accelerating factor, a regulator originally characterized as a plasma membrane shield that circumvents C3b deposition on cell surfaces and prevents C5 activation. They found an augmented T cell response with markedly more IFNγ + and IL-17+ T cell generation in concert with markedly augmented myelin destruction [69]. These findings are not only due to increased IL-12 and IL-23 elaboration by APCs but also to increased T cell expression of the receptors for each of these cytokine [69]. The role of the complement system in IL-23-driven autoimmune neuroinflammation is valuable to study in future projects.

Another important point in our study is the increase of CD11b^high^ CD45^high^ cells in the cerebellum representing activated microglia or macrophages. Expression of the IL23 receptor on macrophages and microglia was described [70, 71], which may enable these cells to directly interact with IL-23. While lymphocytes are early elevated in the course of the EAE in the transgenic mice, it is noteworthy that a significant increase in CD11b^high^CD45^high^ cells representing activated microglia or macrophages, only becomes apparent at a later point, at the end of the observation period of 60 days. This points to the importance of lymphocytes in the early phase of the disease course, while the increase of CD11b^high^CD45^high^ cells seems to be secondary. Persistent activation of microglia has been reported in the chronic phase of EAE with a correlation between activated microglia cells and loss of neuronal synapses [72]. In MS patients, activation of microglia, more often in the progressive form, was found in association with active white matter lesions [73]. Thereby, macrophages and microglia are involved in demyelination and phagocytosis of the degraded myelin [74]. But also anti-inflammatory effects by secreting anti-inflammatory cytokines and promotion of neurogenesis by secreting neurotrophic factors were reported [75, 76].

## Conclusion

Taken together, this study dissects the role of CNS-specific expression of IL-23 in neuroinflammation with quite impressive findings. The data presented here validate IL-23 once more as a crucial factor driving inflammatory processes in the CNS. Especially the aggravated and chronic phenotype of the GF-IL23 mice after immunization with MOG35-55, the multilocular infiltration with high amounts of lymphocytes, especially B cells, the persistent demyelination and the proinflammatory milieu with upregulation of lymphocyte activation markers and co-stimulatory are striking. Among the various immune players elevated, the upregulation of chemokines and the upregulation of the complement system are crucial. The GF-IL23 model proved to be a useful tool to further dissect the impact of IL-23 on neuroinflammatory models.

## Supplementary Information

**Additional file 1 **. **Figure S1** BSA immunized GF-IL23/WT mice show no infiltrates. Routine histological staining (HE) excluded infiltrates in BSA immunized GF-IL23/WT mice (n=5) in the myelon (A, B) and cerebellum (C, D). The images are representative of those from 5 different transgenic/ WT EAE mice on day 22. E The mRNA levels of key proinflammatory marker (myelon/ cerebellum) of BSA immunized GF-IL23/ WT mice (n=5) were normalized to GAPDH. qPCR data detected no significant up- or downregulation with p <0.05 considered to be significant

**Additional file 2 **. **Figure S2** Microglia, astrocyte activation and B cell accumulation in GF-IL23 EAE cerebellum. A-X GF-IL23 mice display microglia, astrocyte activation and B cell accumulation compared to WT mice at day 15, d22 and d33. No microglia, astrocyte activation or infiltration was detected in naïve mice (A-F). Scale bar: 100 μm. The images are representative of those from at least 5 different transgenic/ 5 WT mice d0, d15, d22, d33.

**Additional file 3 **. **Figure S3** mRNA-profile of the cerebellum. Log2 fold change and p value (< 0.05 considered to be significant and in bold letters) of each target of the transcriptome analysis. The transcriptome analysis illustrates the mRNA levels of several surface cell markers, pro-/antiinflammatory markers and complement components of the cerebellum of GF-IL23 and WT mice at d22 and d33 after induction of the EAE. Data were generated from naïve GF-IL23 mice n=6, MOG immunized GF-IL23/WT mice n=4. CD= Cluster of differentiation, CCL= Chemokine (C-C motif) ligand, CCR= C-C chemokine receptor type, CXCL= Chemokine (C-X-C motif) ligand, H2-Eb1= histocompatibility 2, class II antigen E beta, ICAM-1= intercellular adhesion molecule 1, IFNγ= interferon gamma, IL= Interleukin, Foxp3= forkhead box P3, C1-8= complement component 1-8, CFH= complement component factor H

## Data Availability

The datasets used and analyzed during the current study are available from the corresponding author on reasonable request.

## Abbreviations

APC: Antigen-presenting cells
BSA: Bovine serum albumin
C1-8: Complement component 1–8
CCL: Chemokine (C–C motif) ligand
CCR: C–C chemokine receptor type
CD: Cluster of differentiation
CFH: Complement component factor H
CNS: Central nervous system
CSF: Cerebrospinal fluid
CXCL: Chemokine [C–X–C motif] ligand
EAE: Experimental autoimmune encephalomyelitis
Foxp3: Forkhead box P3
GFAP: Glial fibrillary acidic protein
GM-CSF: Colony stimulation factor 2
H2-Eb1: Histocompatibility 2, class II antigen E beta
HE: Hematoxylin and eosin
ICAM-1: Intercellular adhesion molecule 1
IFN: Interferon gamma
IL: Interleukin
IL23R: IL23 receptor
LFB: Luxol fast blue
MOG: Myelin oligodendrocyte glycoprotein
MS: Multiple sclerosis
PBS: Phosphate-buffered saline
Rorc: RAR-related orphan receptor C
Stat: Signal transducer and activator of transcription
T bet: T box expressed in T cells
TGF: Transformation of the growth factor
Th1: T helper 1
Th17: T helper 17
TNF: Tumor necrosis factor
