# Supplementary figures and images for: Astrocyte-specific expression of interleukin 23 leads to an aggravated phenotype and enhanced inflammatory response with B cell accumulation in the EAE model
Source: J Neuroinflammation. 2021 Apr 27;18:101. doi: 10.1186/s12974-021-02140-z (PMC8080359; doi:10.1186/s12974-021-02140-z)

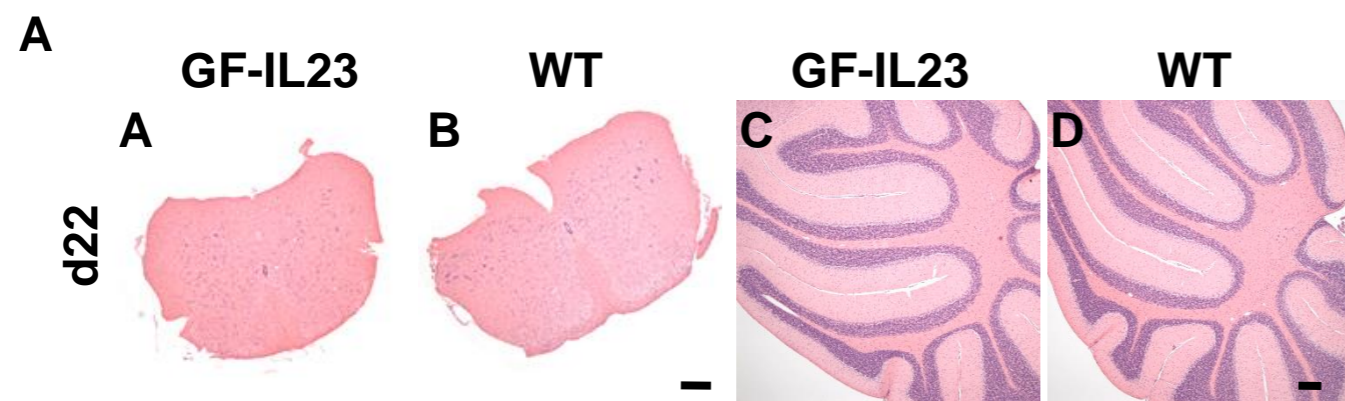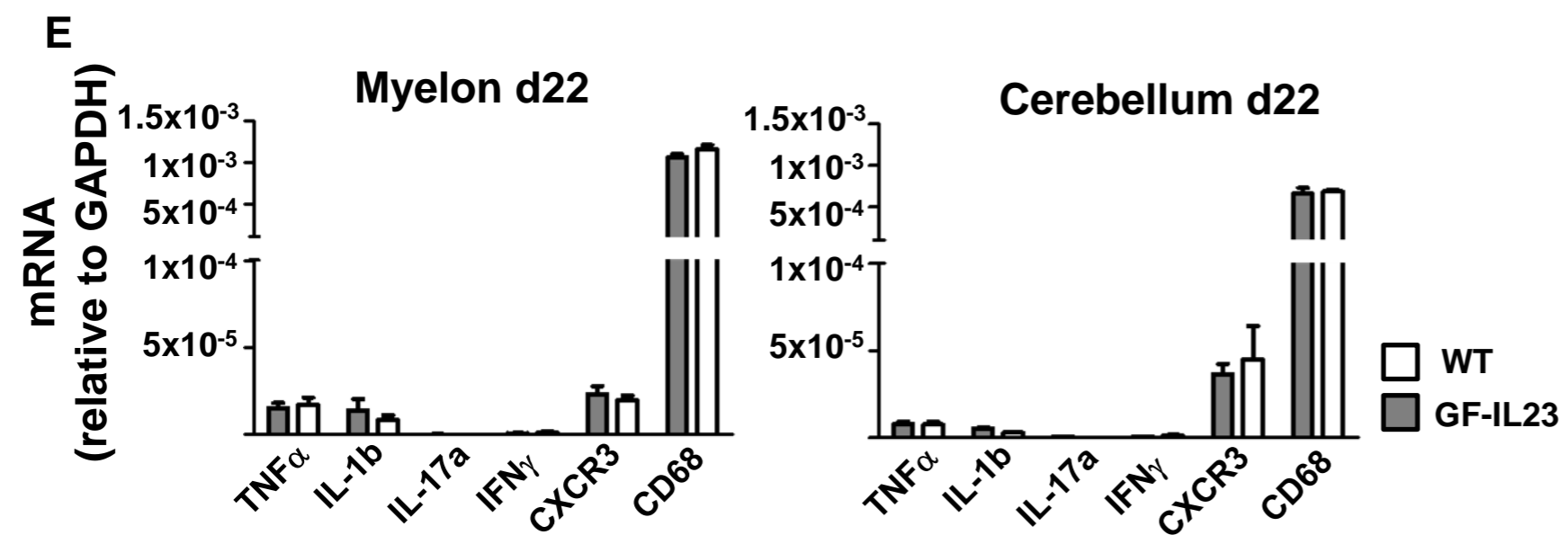

Supplement: Supplementary file 1 — Additional file 1 . Figure S1 BSA immunized GF-IL23/WT mice show no infiltrates. Routine histological staining (HE) excluded infiltrates in BSA immunized GF-IL23/WT mice (n=5) in the myelon (A, B) and cerebellum (C, D). The images are representative of those from 5 different transgenic/ WT EAE mice on day 22. E The mRNA levels of key proinflammatory marker (myelon/ cerebellum) of BSA immunized GF-IL23/ WT mice (n=5) were normalized to GAPDH. qPCR data detected no significant up- or downregulation with p <0.05 considered to be significant [file 12974_2021_2140_MOESM1_ESM.pdf]

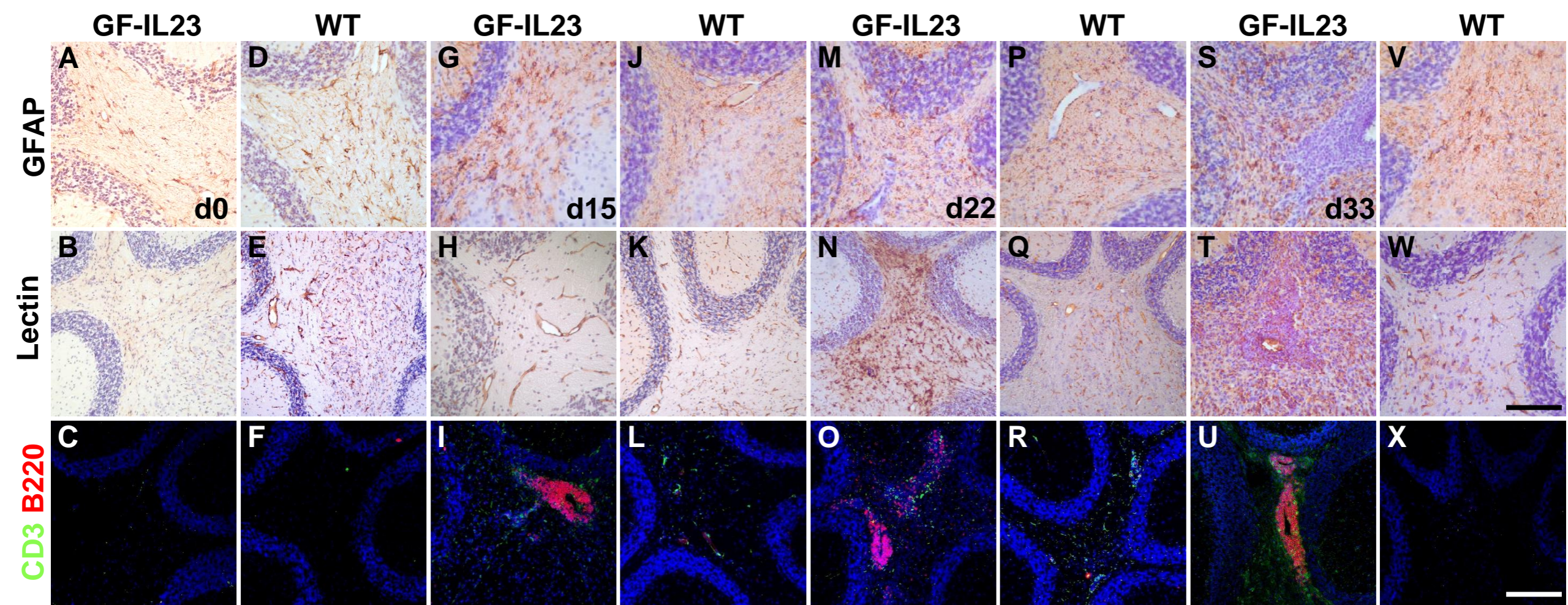

Supplement: Supplementary file 2 — Additional file 2 . Figure S2 Microglia, astrocyte activation and B cell accumulation in GF-IL23 EAE cerebellum. A-X GF-IL23 mice display microglia, astrocyte activation and B cell accumulation compared to WT mice at day 15, d22 and d33. No microglia, astrocyte activation or infiltration was detected in naïve mice (A-F). Scale bar: 100 μm. The images are representative of those from at least 5 different transgenic/ 5 WT mice d0, d15, d22, d33. [file 12974_2021_2140_MOESM2_ESM.pdf]
