# Supplementary material for: Astrocyte-specific expression of interleukin 23 leads to an aggravated phenotype and enhanced inflammatory response with B cell accumulation in the EAE model
Source: J Neuroinflammation. 2021 Apr 27;18:101. doi: 10.1186/s12974-021-02140-z (PMC8080359; doi:10.1186/s12974-021-02140-z)
Supplement: Supplementary file 3 — Additional file 3 . Figure S3 mRNA-profile of the cerebellum. Log2 fold change and p value (< 0.05 considered to be significant and in bold letters) of each target of the transcriptome analysis. The transcriptome analysis illustrates the mRNA levels of several surface cell markers, pro-/antiinflammatory markers and complement components of the cerebellum of GF-IL23 and WT mice at d22 and d33 after induction of the EAE. Data were generated from naïve GF-IL23 mice n=6, MOG immunized GF-IL23/WT mice n=4. CD= Cluster of differentiation, CCL= Chemokine (C-C motif) ligand, CCR= C-C chemokine receptor type, CXCL= Chemokine (C-X-C motif) ligand, H2-Eb1= histocompatibility 2, class II antigen E beta, ICAM-1= intercellular adhesion molecule 1, IFNγ= interferon gamma, IL= Interleukin, Foxp3= forkhead box P3, C1-8= complement component 1-8, CFH= complement component factor H [file 12974_2021_2140_MOESM3_ESM.pdf]

	tg d22 – wt d22		tg d33 – wt d33	
	log2 Fold Change	p value	log2 Fold Change	p value
CD3e	3.37	0.00044	3.57	0.00013
CD4	1.95	0.025	6.31	0.0000003
CD8a	2.34	0.059	7.26	0.000001
CD19	3.23	0.012	7.89	0.0000001
CD11b	1.09	0.061	1.99	0.00064
CD11c	3.01	0.019	4.02	0.0017
CCL5	4.48	0.00023	5.063	0.000032
CCL7	3.14	0.0088	4.71	0.00064
CCL8	3.21	0.0084	3.57	0.0033
CCR1	-3.15	0.027	1.29	0.24
CCR6	2.67	0.022	4.95	0.000026
CCR7	1.45	0.21	5.09	0.000049
CXCL9	3.69	0.0095	3.86	0.0066
CXCL10	3.34	0.027	3.41	0.024
CXCL13	4.17	0.015	6.51	0.00013
CXCR3	3.31	0.022	3.67	0.00093
CD20	1.21	0.51	5.72	0.0029
CD25	1.61	0.13	3.68	0.0013
CD27	2.29	0.13	5.24	0.00013
CD40	1.93	0.0073	3.84	0.0000010
CD44	1.15	0.11	2.55	0.00036
CD68	1.82	0.021	3.37	0.000019
CD69	2.34	0.22	4.53	0.028
CD80	2.05	0.47	3.84	0.18
CD86	3.03	0.00031	3.41	0.000024
H2-Eb1	3.35	0.0049	4.05	0.00066
Icam1	2.39	0.000071	2.41	0.000061

	tg d22 – wt d22		tg d33 – wt d33	
	log2 Fold Change	p value	log2 Fold Change	p value
IFNγ	3.05	0.21	4.77	0.043
IL-1a	0.31	0.87	3.68	0.041
IL-1b	2.16	0.049	2.76	0.012
IL-2	0.53	0.56	1.38	0.14
IL-5	0.00	1.00	0.00	1.00
IL-6	1.91	0.73	0.81	0.88
IL-7	-0.041	0.99	3.89	0.15
IL-10	0.00	1.00	3.44	0.46
IL-17a	3.19	0.49	2.49	0.59
IL-17f	0.00	1.00	-2.43	0.65
IL-21	1.35	0.69	1.86	0.58
IL-22	0.31	0.82	-0.99	0.41
Foxp3	2.76	0.075	5.26	0.000096
TGFβ	2.11	0.00035	1.96	0.00074
TNFα	2.47	0.023	3.51	0.0018
C6	2.31	0.38	4.41	0.092
C7	0.97	0.81	-3.11	0.44
C8a	0.34	0.95	0.00	1.00
C8b	0.076	0.99	0.81	0.87
C8g	-0.42	0.68	0.61	0.49
C1qb	2.19	0.00029	2.11	0.00052
C1qc	2.12	0.0036	3.19	0.000012
C2	1.87	0.0019	2.22	0.00019
C4a	1.11	0.28	3.83	0.0055
C4b	2.86	0.000073	2.55	0.00041
C3	2.55	0.017	3.71	0.00052
CHF	1.29	0.011	0.96	0.054
